# Risk factors for benign prostatic hyperplasia: a comprehensive review

**DOI:** 10.1590/1806-9282.20250343

**Published:** 2025-07-07

**Authors:** Mário Henrique Bitar Siqueira

**Affiliations:** 1 University of Toronto, Division of Urology – Toronto, Canada.

## INTRODUCTION

Benign prostatic hyperplasia (BPH) is increasingly prevalent with advancing age, affecting approximately 50% of men aged over 50 years and rising to over 80% by the eighth decade of life^1^. Histological prevalence at autopsy is estimated to be between 50 and 60% in men in their 60s, increasing to 80–90% in those aged over 70 years^2^. Despite its widespread occurrence, the relationship between risk factors and disease development remains poorly understood, and many studies rely on the broad definitions of BPH, complicating the identification of ­definitive risk factors^3^.

Heritability plays a significant role in BPH, with family and twin studies estimating that genetic factors contribute to 39–72% of cases^4^. A validation cohort identified significant genetic variants associated with BPH diagnosis or treatment, including those in the progesterone receptor, RBMS1 (RNA/DNA binding in cell cycle/death), MPPED1 (metallophosphoesterases), and NPAP1 (tissue-specific imprinting, spermatogenesis). Genome-wide association studies (GWAS) have highlighted additional variants linked to BPH, such as SYN3 (synaptogenesis and neurotransmission), GCLC (glutathione synthesis), UNC13A, DCC, BTBD3 (dendritic organization), and ELVOVL3^5^.

Using a comprehensive suite of analytical methods, other studies have identified BTN3A2 and C4A as key genetic contributors to BPH pathogenesis and further delineated the cg14345882-BTN3A2-BPH pathogenic pathway. Leveraging druggable gene data, we identified 30 potential therapeutic targets, including BTN3A2 and C4A. Additionally, Mendelian randomization (MR) analysis of immune pathways underscored the role of immune cell surface molecules and the inflammatory cytokine (interleukin [IL]-17) as significant mediators in BPH development^6^.

These findings indicate that genetic factors contribute to approximately 60% of the phenotypic variation in BPH^7^. However, an in-depth exploration of modifiable risk factors is necessary to better understand their role in disease progression. This review aims to critically analyze the current scientific evidence on these factors.

## METHODS

A comprehensive literature review was conducted to explore the association between BPH and various modifiable risk ­factors. Relevant peer-reviewed journal articles were identified through comprehensive searches in PubMed, Scopus, Web of Science, and Google Scholar, covering publications from 2014 to 2024. Studies were included if they were published in English or Spanish and examined the pathophysiology of BPH and its associated risk factors. The exclusion criteria comprised studies unrelated to BPH or those focusing on other medical conditions. The search strategy utilized key terms such as "benign prostatic hyperplasia," "risk factors and BPH," and "lower urinary tract symptoms." Articles were screened based on title and abstract, with eligible studies undergoing a full-text review. Ethical approval was not required, as the review relied solely on publicly available data.

### Risk factors

#### Diet

The influence of nutrients on BPH remains somewhat unclear, but several studies provide valuable insights. High protein intake, particularly from animal sources, has been positively associated with an increased risk of BPH^8^. Conversely, other studies have supported the idea that a diet rich in ­phytochemicals—such as those found in whole grains, fruits, vegetables, and nuts—could reduce the likelihood of developing BPH^9^. In a multivariable logistic regression study, fruit and vegetable consumption was significantly linked to a lower risk of BPH. Participants with insufficient fruit intake were 21 times more likely to develop BPH compared to those with adequate fruit consumption^10^.

The protective effects of vegetables, however, appear to vary depending on specific nutrient compositions. For instance, an increase of 0.01 μmol/L in serum retinyl esters was found to be associated with a 2% increase in BPH risk. Similarly, elevated vitamin E levels correlated with a higher risk of BPH, particularly in men over 60 years old^11^. Niacin intake has also been implicated as a risk factor, with a study involving 700 men revealing that those in the highest tertile of dietary niacin intake were more than twice as likely to develop BPH compared to those in the lowest tertile (OR 2.34, 95%CI 1.24–4.42)^12^.

Beyond individual nutrients, overall dietary patterns also appear to influence BPH risk. Pro-inflammatory diets characterized by a high consumption of processed meats, refined sugars, and trans fats have been linked to an increased likelihood of BPH. After adjusting for potential confounders, a higher Dietary Inflammatory Index (DII) was positively associated with BPH risk, further supporting the role of inflammation in disease progression. These findings highlight the importance of dietary factors in the pathophysiology of BPH and suggest potential avenues for dietary modifications to mitigate risk^13^.

### Metabolic syndrome

Metabolic syndrome (MetS) and its associated pathological factors have been implicated in increased prostate volume (PV), which may accelerate disease progression^14^. Preclinical and clinical evidence suggests that modifiable, age-related metabolic abnormalities, including MetS, play a key role in the development and progression of BPH-lower urinary tract symptoms (LUTS)^15,16^. A meta-analysis of 16 studies involving 1,895 patients with BPH, including 1,224 with MetS, demonstrated that individuals with MetS had significantly greater total PV (weighted mean difference [WMD]=10.15 mL; 95%CI 7.37–12.93) and higher serum prostate-specific antigen (PSA) levels (WMD=0.53 ng/mL; 95%CI 0.17–0.88) than those without MetS^17^. Additionally, prostate growth rates were significantly elevated in MetS patients, with an annual increase of 47% in those with Type 2 diabetes mellitus, 17% in those with hypertension, 36% in obese individuals, 31% in men with low high-density lipoprotein (HDL) cholesterol, and 28% in those with elevated fasting insulin levels^18,19^.

Beyond prostate enlargement, MetS and its individual components have been identified as significant risk factors for various urological conditions. Given these associations, lifestyle interventions targeting metabolic health may offer a preventive strategy against the onset and progression of these disorders^20^.

#### Obesity

Obesity is a well-established risk factor for multiple urological conditions, including BPH. A body mass index (BMI) >30 kg/m² has been linked to an increased risk of nephrolithiasis, kidney cancer, overactive bladder, male hypogonadism, BPH, and erectile dysfunction^21^. A study of 6,253 men over 40 years old, with 4,321 follow-ups in 2018, demonstrated a positive correlation between high relative fat mass (RFM) and the prevalence of LUTS and BPH^22^.

Genetic evidence further supports the association between central obesity and BPH. Data from the UK Biobank indicate that genetically predicted higher waist circumference and waist circumference adjusted for BMI are associated with an increased risk of BPH. The combined odds ratios (ORs) for BPH were 1.24 (95%CI 1.07–1.43, p=0.0045) for waist circumference, 1.26 (95%CI 1.11–1.43, p=0.0004) for waist circumference adjusted for BMI, and 1.08 (95%CI 1.01–1.17, p=0.0175) for BMI^23^. Additionally, an MR study confirmed that genetically predicted waist circumference (OR 1.26, 95%CI 1.11–1.43, p=0.0004) is statistically significantly associated with an elevated risk of BPH^24^.

#### Hyperglycemia/hyperinsulinemia

Insulin resistance and hyperinsulinemia, the key components of MetS, have been implicated in the pathogenesis of BPH and LUTS. In vitro studies suggest that hyperinsulinemia promotes prostatic epithelial cell proliferation. Clinically, patients with serum insulin levels exceeding 13 mU/L exhibit larger PVs and a higher annual BPH growth rate compared to those with lower insulin levels^25^.

The association between hyperinsulinemia and BPH/LUTS may be mediated by increased sympathetic nerve activity, which contributes to greater prostate smooth muscle tone and bladder outlet obstruction^26^. This elevated outlet resistance can lead to obstructive urinary symptoms, which may progress to irritative symptoms over time^27^. McVary et al. further highlighted a correlation between autonomic nervous system ­hyperactivity, LUTS severity, and prostate enlargement in a cohort of 38 men^28^. Additionally, the trophic effects of elevated insulin levels in diabetic patients may contribute to prostate growth.

Dysregulation of the insulin-like growth factor (IGF) axis has also been implicated in BPH pathogenesis. Due to structural similarities between insulin and IGF, insulin can bind to IGF receptors on prostate cells, activating pathways that promote cell proliferation. Furthermore, insulin may upregulate genes and proteins involved in sex hormone metabolism, altering the prostatic hormonal environment^29^.

Chronic inflammation associated with hyperglycemia may further contribute to BPH/LUTS development^30^. Clinically, hyperglycemia and insulin therapy have been linked to an increased risk of BPH surgery, suggesting that untreated hyperglycemia and exogenous insulin use may be significant risk factors for BPH progression^27^.

Conversely, a prospective population-based cohort study in Michigan (n=2,226) found no significant association between diabetes—regardless of treatment status—and increased PV (>30 cm³) or elevated PSA levels (>2.5 ng/mL) after ­adjusting for age and race. These findings suggest that diabetes and poor glycemic control may influence LUTS through dynamic bladder dysfunction rather than direct prostate enlargement^31^.

#### Dyslipidemia

In a study comprising two cohorts—one with hyperlipidemia (n=8,965) and another without hyperlipidemia (n=26,895)—individuals with hyperlipidemia exhibited a significantly higher risk of developing BPH. After propensity score adjustment, the hyperlipidemia cohort had a markedly greater risk compared to the non-hyperlipidemia cohort (hazard ratio [HR]=1.73, 95%CI 1.63–1.83, p<0.001)^32^.

Further meta-regression analysis revealed that PV differences were more pronounced in obese patients, older individuals, and those with low serum HDL cholesterol levels^33^. Among MetS components, lower HDL and higher triglyceride levels were strongly correlated with increased prostatic inflammation, driven by IL-8 secretion in response to oxidized LDL and insulin. Additionally, a recent cross-sectional study in a BPH/LUTS cohort demonstrated that men with moderate-to-severe LUTS had over a five-fold increased likelihood of a Framingham risk score of ≥10%^34^.

### Cardiovascular disease

In Chinese men, BPH has been associated with an increased risk of cardiovascular disease (CVD), heart disease, and stroke, particularly among those aged <60 years^35^. Further supporting this association, additional studies have reported an increased risk of angina pectoris, heart failure, and atrial fibrillation^36^. These findings suggest that BPH may contribute to a spectrum of cardiovascular conditions, potentially through shared pathophysiological mechanisms.

Emerging evidence indicates that hormonal and inflammatory alterations in BPH patients may not only exacerbate LUTS but also contribute to metabolic and autonomic dysregulation, collectively increasing CVD risk^37^. Additionally, nocturia—a common symptom of BPH—may further heighten cardiovascular risk by disrupting sleep patterns and circadian rhythms. This disruption reduces antidiuretic hormone (ADH) secretion and testosterone levels, both of which are associated with MetS and cardiovascular dysfunction^38^.

A potential causal relationship between BPH and CVD has also been proposed. While BPH appears to be a risk factor for coronary heart disease (CHD) and myocardial infarction (MI), some studies paradoxically suggest a protective effect against stroke. However, reverse causality has not been observed, and further research with larger cohorts is necessary to clarify these associations^39^.

### Sleep disorders

Several studies suggest that adequate sleep duration may reduce the risk of developing BPH^40^. An Indian study involving 453 men found that poorer sleep quality was significantly associated with a higher incidence of BPH, while higher sleep quality scores were linked to a lower risk, particularly in middle-aged and older men^41^.

Additionally, the prevalence of obstructive sleep apnea (OSA) has been implicated in the development of BPH, especially in younger men^42^. An MR analysis using genetic data further validated the association between genetically predicted insomnia symptoms and an increased risk of BPH^43^.

Despite growing evidence, the exact relationship between BPH, LUTS, and circadian rhythm disruption remains unclear. Potential mechanisms include pro-inflammatory states, MetS, and hormonal dysregulation, all of which may be influenced by circadian disturbances^44^. These findings suggest that improving sleep quality and addressing insomnia may serve as important strategies for BPH prevention.

### Uric acid

The relationship between uric acid (UA) levels and BPH remains a topic of debate. Some studies indicate a positive correlation, particularly among younger gout patients (aged 41–60 years)^45^. Notably, men who used allopurinol demonstrated a decreased risk for multiple BPH-related protective associations that ­disappeared after a 1–2-year lag period, suggesting a potential time-dependent effect^46^.

A study involving 2,845 participants (531 with BPH and 2,314 controls) found that after adjusting for confounders, the risk of developing BPH decreased by 18% for every 100 μmol/L increase in UA levels^47^. Subgroup analyses revealed that this inverse association was more pronounced in participants <60 years old, non-Hispanic Whites, former smokers, heavy drinkers, non-diabetics, and those with hypertension^48^. These findings suggest that demographic and health-related factors may influence the association between UA and BPH, necessitating further research to elucidate the underlying mechanisms.

## CONCLUSION

While the relationship between MetS, diet, and BPH is complex, existing evidence suggests that high animal protein intake, obesity, and pro-inflammatory diets may elevate BPH risk. Conversely, diets rich in fruits, vegetables, and phytochemicals, as well as metabolic regulation strategies such as ­hyperinsulinemia ­management, may confer protective effects. Additionally, comorbidities such as obesity, diabetes, and hyperlipidemia appear to accelerate BPH progression, underscoring the importance of a comprehensive approach to prevention and management. Future research should aim to clarify the mechanistic pathways linking metabolic, cardiovascular, and sleep-related factors to BPH, with an emphasis on targeted interventions.

## Data Availability

The datasets generated and/or analyzed during the current study are available from the corresponding author upon reasonable request.
